# Malaria Prevention in Imposed War 1980–1988

**Published:** 2019

**Authors:** Ali MEHRABI TAVANA

**Affiliations:** Health Management Research Center, Baqiyatallah University of Medical Sciences, Tehran, Iran

## Dear Editor-in-Chief

Malaria is a vector borne disease caused by protozoan parasite of the *Plasmodium* (include *P. falciparum, P. vivax, P. ovale*, or *P. malariae*). It is mainly transmitted by female *Anopheles* mosquitoes distributed in the nature mostly in tropical and semi-tropical regions. Of course, the disease could be transmitted from infected patients to healthy people via blood transfusion (1), organ transplantation (2), or in IV drug abusers (3) and even from the infected mother to the child via fetus (4).

Nowadays, many malaria parasites and their vector have become resistant to many anti malaria drugs and insecticides. Malaria is still a main problem in the world in particular in different developing countries (5). An estimated 214 million cases of malaria occurred worldwide and 438,000 people died in 2015 (6).

From 25 species of *Anopheles* found in Iran, 8 species of *A. stephensi, A. fluviatilis, A. culicifacies, A. pulcherimus, A. d'thali, A. superpictus, A. sacharovi and A. maculipennis* are considered to be malaria vectors (7). In addition, the *P. falciparum* and *P. vivax* are more prevalent species of *Plasmodiain* in Iran. Different approaches were adopted to eradicate the disease in the last century by collaboration of national and international organizations (7).

The disease had occurred in epidemic form in different war and conflicts in the past. The history is fraught with the deaths of those who died of malaria in the wars. The imposed war happened in the region in 1980–1988, where the disease was endemic in both countries (Iran-Iraq). During wartime, malaria was a public health concern not only for the Iranian Ministry of Health but also for health care providers from different Iranian military troops including Iranian Army and Islamic revolutionary guard crops.

For prevention and control of the disease, many actions were taken (8) with collaboration with different health sectors across the country including;

- **Health education:** Health education to the soldiers as face to face to them and using different teaching methods (distribution of pamphlets, posters and demonstration of film related the disease.
- **Chemoprophylaxis**: Prevention of the disease was made possible by chemoprophylaxis (using pyrimethamine 25 mg weekly based on guideline which was prepared by the Iranian Ministry of Health.
- **Insect control:** By use of the different insecticides such as (DDT, malathion and baygon for indoor residual spraying including resting places for soldiers under scientific supervision of Iranian ministry of health and their relevant administration (i.e. diseases management control and using top standard sprayer pumps (Hudson) by trained workers for controlling the vector at least twice a year (March and September) during wartime. Larviciding using chemicals (oiling or Abate) was also used in the parts of war zones too.
- **Case finding:** Very active case finding was done among soldiers before arriving at the battle fields.
- **Environment sanitation:** With consideration of measures to improve the environment and oil spill in the ponds within the frontier, attempts have been made to reduce the *Anopheles* mosquito laying.
- **Protective measures:** By distribution of insect repellents such as 'Sangar ointment' and similar compounds' among combatants to reduce the bite rates by protecting their face and hands overnight.

Ordinary bed net was also available for some time, but treated insecticide bed net was not available in wartime for everyone. In addition, the control measured not only affected to reduce malaria cases but also reduce the cases of other vector borne diseases (i.e. sand fly fever) which were endemic in the same region too (9–10).

During the war period, the disease has been endemic in some areas of northern and southeastern Iraq (adjacent to the borders of Iran). Therefore, its control has been of particular importance and its control has been expressed. In spite of endemic situation, the disease in the region between Iran-Iraq boarders only 92 malaria cases were reported during 1980–1988. Malaria was well controlled in wartime by taking different health measures which is mentioned earlier. The inter-sector collaboration to control the disease must continue by the monitoring and surveillance in order to prevent a possible return of the disease.
